# Socioeconomic privilege and political ideology are associated with racial disparity in COVID-19 vaccination

**DOI:** 10.1073/pnas.2107873118

**Published:** 2021-07-29

**Authors:** Ritu Agarwal, Michelle Dugas, Jui Ramaprasad, Junjie Luo, Gujie Li, Guodong (Gordon) Gao

**Affiliations:** ^a^Department of Decision, Operations, and Information Technologies, Robert H. Smith School of Business, University of Maryland, College Park, MD 20742;; ^b^Center for Health Information and Decision Systems, Robert H. Smith School of Business, University of Maryland, College Park, MD 20742

**Keywords:** racial disparity, COVID-19, social determinants of health, vaccination

## Abstract

Vaccine uptake is critical for mitigating the impact of COVID-19 in the United States, but structural inequities pose a serious threat to progress. Racial disparities in vaccination persist despite the increased availability of vaccines. We ask what factors are associated with such disparities. We combine data from state, federal, and other sources to estimate the relationship between social determinants of health and racial disparities in COVID-19 vaccinations at the county level. Analyzing vaccination data from 19 April 2021, when nearly half of the US adult population was at least partially vaccinated, we find associations between racial disparities in COVID-19 vaccination and median income (negative), disparity in high school education (positive), and vote share for the Republican party in the 2020 presidential election (negative), while vaccine hesitancy is not related to disparities. We examine differences in associations for COVID-19 vaccine uptake as compared with influenza vaccine. Key differences include an amplified role for socioeconomic privilege factors and political ideology, reflective of the unique societal context in which the pandemic has unfolded.

Vaccines are essential to forging a path out of the COVID-19 pandemic. At the same time, systemic racial inequities in the United States pose the risk of disparities in vaccination rates among vulnerable populations. This is especially important as COVID-19 has disproportionately impacted racial minorities (1, 2). Racial disparities in COVID-19 vaccination rates represent a salient example of racism’s “serious threat to public health” (3), a role recently recognized by the director of the Centers for Disease Control and Prevention. Prior studies have documented significant racial disparities in vaccination rates for other diseases, with the most persistent differences observed between Blacks and Whites (4). Explanations for such disparities abound, including structural racism, medical mistrust, and individual vaccine hesitancy (5).

We investigate the role of five dimensions of social determinants of health, conditions that “affect a wide range of health, functioning, and quality-of-life outcomes and risks” (6), in vaccination disparities. We estimate the impact of economic stability, education access and quality, health care access and quality, neighborhood and built environment, and social and community context on racial disparities in COVID-19 vaccination rates. Vaccine disparity is measured as the rates of those receiving at least one vaccination dose for Whites less that for Blacks, where a higher value suggests greater disparities for Blacks. Our analysis is at the county level, and we control for vaccine hesitancy and proportion of a county’s population that is Black. To shed light on the unique context of COVID-19, we conduct the same analysis for influenza vaccination rates. The two diseases are distinct in that COVID-19 is deadlier, and COVID-19 vaccines were developed rapidly and deployed with limited supply in the early days of their temporary-use authorization (7).

## Results

**Fig. 1.**
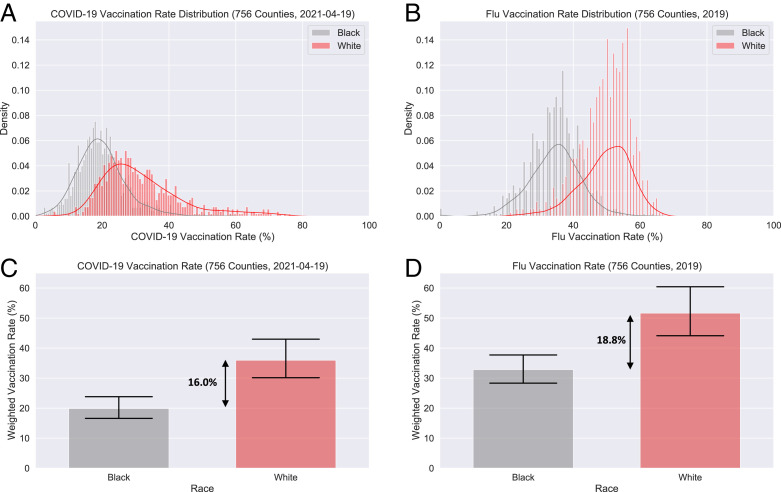
(*A*) Distribution of county-level COVID-19 vaccination rates by race (19 April 2021). (*B*) Distribution of county-level flu vaccination rates among Medicare beneficiaries for the year 2019 by race. (*C*) Weighted average of Black and White rates for COVID-19 vaccination and their disparity. (*D*) Weighted average of Black and White rates for flu vaccination and their disparity. Error bars represent 95% confidence intervals.

Our analysis includes 756 US counties, covering over 170.6 million lives, representing 51.5% of the US population. The data are from 19 April 2021, when about half of the adult US population had received at least one dose of a COVID-19 vaccine and the vaccines were made available to all US adults. Fig. 1 visualizes COVID-19 and flu vaccine disparities across counties in our sample.

**Fig. 2.**
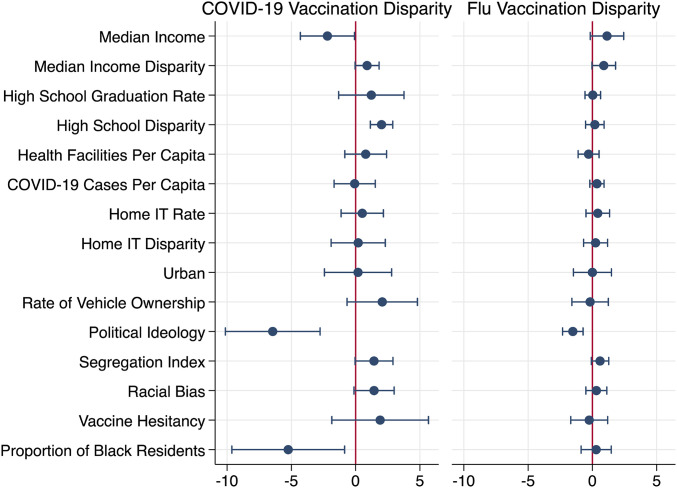
Contrast of regression coefficients for indicators of social determinants of health for COVID-19 and flu vaccine disparities (*n* = 756). Percentage point change in disparities associated with one SD increase in predictor variables. Error bars represent 95% confidence intervals.

Results are in Table 1 and Fig. 2. For each variable where data are available by race we model the racial disparity (median income, education, and information technology [IT] in the home) in addition to the level. Results from similar analyses for data on 27 March, 7 April, and 20 May are consistent (see *SI Appendix*). We standardize all continuous variables.

**Table 1.** Regression estimates of relationship between social determinants and COVID-19 and flu vaccination disparities

Variable category	Variable	CVD	FVD
Economic stability	Median income	−2.20* (0.99)	1.14^†^ (0.61)
	Median income disparity	0.89^†^ (0.44)	0.88^†^ (0.43)
Education access and quality	High school graduation rate	1.22 (1.19)	0.03 (0.28)
	High school disparity	2.01*** (0.41)	0.19 (0.34)
Healthcare access and quality	Health facilities per capita	0.78 (0.76)	−0.30 (0.38)
	COVID-19 cases per capita	−0.08 (0.75)	0.35 (0.26)
Neighborhood and built environment	Home IT rate	0.51 (0.77)	0.42 (0.43)
	Home IT disparity	0.20 (0.99)	0.25 (0.44)
	Urban	0.19 (1.23)	0.001 (0.70)
	Rate of vehicle ownership	2.07 (1.28)	−0.18 (0.67)
Social and community context	Political ideology	−6.45** (1.73)	−1.52*** (0.37)
	Segregation index	1.43^†^ (0.69)	0.60^†^ (0.32)
	Racial bias	1.43^†^ (0.73)	0.31 (0.38)
Constant		8.286*** (1.44)	13.46*** (0.92)

Covariates: vaccine hesitancy and proportion of Black residents (see *SI Appendix*, *Methods for Regression Analysis*). Each model includes data for 756 counties. Models are estimated with state dummies, robust SEs clustered at state level and weighted by county population. All continuous predictors are standardized. R-squared CVD = 0.67; R-squared FVD = 0.46. ^†^*P* < 0.10, *P < 0.05, **P < 0.01, ***P < 0.001.

### COVID-19 Vaccination Disparities (CVD).

The weighted average CVD across counties is 16.0 percentage points (White rate: mean [*M*] = 35.9%, SD = 11.06; Black rate: *M* = 19.9%, SD = 6.11). Vaccine hesitancy, often considered a driver of disparities (5), is not significant in the weighted ordinary least squares (OLS) estimation. Indicators of four of five social determinants of health, excluding healthcare access and quality, are significantly associated with the CVD.

Vaccination disparities are negatively associated with median income (estimate [Est.] = −2.20, SE = 0.99). Disparity in high school graduation is associated with increased CVD (Est. = 2.01, SE = 0.41). Home IT rate (a measure of internet penetration and availability of devices such as computers and mobile phones) and home IT rate disparity are uncorrelated with vaccination disparities, as is vehicle ownership rate. Results for social and community context variables indicate that political ideology (proportion of the 2020 presidential election votes for the Republican candidate) is strongly and negatively associated with CVD (Est. = −6.45, SE = 1.73). Finally, proportion of a county’s population that is Black, a covariate in the model, is negatively associated with CVD (Est. = −5.24, SE = 2.06).

### Contrast to Flu Vaccination Disparities (FVD).

The weighted average FVD across counties in our analysis is 18.8 percentage points (White rate: *M* = 51.6%, SD = 6.48; Black rate: *M* = 32.8%, SD = 6.52). In contrast to CVD, overall median income is marginally positively associated with increased FVD (Est. = 1.14, SE = 0.61), as is disparity in median income (Est. = 0.88, SE = 0.43). Disparity in high school graduation is unrelated to FVD (Est. = 0.19, SE = 0.34), unlike CVD. Notably, political ideology is negatively associated with both, but the relationship is weaker for FVD (Est. = −1.52, SE = 0.37). The proportion of Black population in a county is unrelated to FVD, unlike CVD.

The CVD results for disparity in median income, high school education, political ideology, and proportion of Black population are robust when controlling for the proportion of people 75 y of age and older (given vaccine prioritization), between 15 and 74 y (given ages eligible for vaccines), and for FVD (a significant predictor of CVD). These findings are also consistent for CVD across a variety of specifications and ordinate measures of disparity (i.e., the ratio of vaccination rates and scaling the absolute difference by average vaccination rate). Median income and overall flu vaccination rate become nonsignificant in certain other robustness checks.

## Discussion

We find that CVD is associated with median income, education, and political ideology. In the face of an unprecedented pandemic (8), with significant resources invested in educating the public and distributing vaccines, these racial disparities persist without signs of abating over a 3-wk (27 March to 19 April 2021) period of a massive national vaccination campaign with a doubling of the vaccination rate (see *SI Appendix*). To the extent that another pandemic is almost inevitable, our findings underscore the importance of spotlighting the inequity in vaccination rates between Blacks and Whites.

Many of these variables have been associated with racial disparities in health in different contexts (3, 9). A clear theme in our results is the centrality of socioeconomic privilege and political ideology. Disparities in high school graduation rates were key drivers of CVD: A county in the 75th percentile of education disparity has a 10.7 percentage point difference in high school graduation rates between White and Black residents, corresponding to an estimated increase in CVD of 2.7 percentage points compared to a county with no high school disparities. This represents a substantial increase given the average CVD rate of 16.0 percentage points in our dataset.

Higher overall median incomes were associated with lower CVD. The difference between a county in the 75th percentile ($64,081) versus the 50th percentile ($54,517) of median income was associated with a 1.3 percentage point drop in CVD. With public health departments leading vaccine rollout coordination, this finding may speak to the additional resources available to higher income counties.

Republican vote share is significantly and negatively associated with CVD and FVD, with a stronger association for CVD, throwing the divisive societal and social media discourse surrounding the pandemic into sharp focus (10). In a February 2021 poll, Republicans expressed a significantly lower willingness to be vaccinated than Democrats (10). Even controlling for vaccine hesitancy, this is reflected in our finding that Republican vote share was strongly associated with less CVD, with a 1 percentage point decrease in CVD for each 2.5 percentage point increase in Republican vote share.

Counties with a greater proportion of Black residents have less disparity in COVID-19 vaccination rates. This pattern emerged despite inclusion of the cumulative COVID-19 cases in a county, controlling for potential disproportionate impact of COVID-19 in these communities (1, 2).

These patterns emerge after controlling for overall vaccine hesitancy in a county, challenging a popular media narrative that foregrounds Black hesitancy, triggered by mistrust in the medical establishment and a history of medical racism (11), as a root cause of disparities in vaccination. Our findings suggest that this might not be the case but rather that the observed disparities are a result of socioeconomic and political factors. Indeed, polls from February 2021 show that Blacks are more willing to get vaccinated than they were in November 2020 (10).

## Implications

With vaccines reaching a point of supply outpacing demand, our findings have implications for vaccine distribution policy, outreach efforts, and patient education and awareness, highlighting areas where special effort is needed to overcome the barriers identified, through access to resources and dissemination of accurate information. Failure to address these structural barriers poses the dual risks of additional lives lost and a significant slowdown in progress toward ending the COVID-19 pandemic or combatting similar future outbreaks. State health departments are not consistently reporting vaccination data by race. Our study underscores a major hurdle in eliminating disparities—the limited availability of granular information to measure disparities and make evidence-based policy decisions on resource allocation and outreach.

## Materials and Methods

COVID-19 vaccination data were collected from state public health department websites (see *SI Appendix*). Flu vaccination data were collected from the Centers for Medicare and Medicaid Services and reflect rates among Medicare beneficiaries (12). Demographic information comes from the Population Estimates table and American Community Survey via the Census Bureau (13). Vaccine hesitancy represents an estimate of the proportion of the county’s residents who “would probably not” or “definitely not” receive the vaccine once available, based on responses to a Household Pulse Survey (17 to 29 March 2021) and were collected from the Office of the Assistant Secretary for Planning and Evaluation at the Department of Health and Human Services (14). Weighted OLS regression analysis included variables winsorized at the 5th and 95th percentile, state dummy variables, and robust SEs clustered at the state level.

## Supplementary Material

Supplementary File

## Data Availability

Data have been deposited in GitHub (https://github.com/CHIDS-UMD/Covid19-Vaccination-Race-Disparity-publish) (15).
