# Supplementary material for: Socioeconomic privilege and political ideology are associated with racial disparity in COVID-19 vaccination
Source: Proc Natl Acad Sci U S A. 2021 Jul 29;118(33):e2107873118. doi: 10.1073/pnas.2107873118 (PMC8379950; doi:10.1073/pnas.2107873118)
Supplement: Supplementary File [file pnas.2107873118.sapp.pdf]

PNAS
www.pnas.org

Supplementary Information for

Socioeconomic Privilege and Political Ideology are Associated with Racial Disparity in COVID-19 Vaccination

Ritu Agarwal, Michelle Dugas, Jui Ramaprasad, Junjie Luo, Gujie Li, Guodong (Gordon) Gao

*Ritu Agarwal
Email: ragar@umd.edu

This PDF file includes:

Supplementary text
SI References

Supplementary Information Text

Outcome Variables

COVID-19 Vaccination. We compiled data from the official websites of Departments of Health in each state, identifying 16 states that provide detailed vaccination data by race at the county level. County-level vaccination data from the 16 states were collected at different points in time, including March 27, April 7, April 19, and May 20, 2021. Where possible, data collection was automated via web scraping; else we utilized manual procedures. We report findings from the April 19 data in the main text, and for the March 27, April 7, and May 20 data as robustness checks in this supplement.

The following states are included in our sample with total number of counties in our regression analysis and the populations for those counties in parentheses, based on 2019 estimates: California (43 counties; 39,089,081 residents) (1), Illinois (41 counties; 11, 410,266 residents) (2), Indiana (37 counties; 5,312,656 residents) (3), Maine (8 counties; 1,011,483 residents) (4), New Jersey (21 counties; 8,882,190 residents) (5), New York (48 counties; 18,793,377 residents) (6), North Carolina (43 counties; 8,564,283); (7), Ohio (57 counties; 10,572,524 residents) (8), Oregon (11 counties; 3,218,739 residents) (9), Pennsylvania (40 counties; 11,656,636 residents) (10), South Carolina (46 counties; 5,148,714 residents) (11), Tennessee (62 counties; 6,107,482 residents) (12), Texas (137 counties; 26,705,914 residents) (13), Virginia (111 counties; 8,209,445 residents) (14), West Virginia (23 counties; 1,271,620 residents) (15), and Wisconsin (28 counties; 4,692,974 residents) (16). In total, 756 counties are included in our regression analysis, representing over 170.6 million Americans.

These states do not treat the intersection of race and ethnicity consistently, precluding distinctions between vaccination rates of non-Hispanic and Hispanic populations of White and Black races in our analysis. Most states report vaccination data by considering *only* race and not ethnicity. In such cases, the number of vaccinations administered to White (or Black) people may be of any ethnicity. Six states (California, Illinois, New Jersey, Oregon, Texas, and Virginia) report vaccination information by race while taking ethnicity into account, reporting information for White, Black, and Hispanic/Latino populations independently. In these cases, it is assumed that vaccination numbers for White (or Black) people consider only those who are non-Hispanic. We take these differences into account when defining our vaccination disparity outcome, as described below.

COVID-19 Vaccination Disparity Operationalization. Consistent with the literature on racial disparities (17–20), we define COVID-19 vaccination disparity (CVD) as the difference in the COVID-19 vaccination rate between White and Black residents in a county, where vaccination rates are based on the total population of a given race in a county. Population denominators were collected at the county-level by race from the Census Bureau's County Population by Characteristics tables, which contain Vintage 2019 county population estimates by demographic characteristics (21).

To construct the CVD measure, we consider the people who have received at least one dose of COVID-19 vaccine at the time of data collection. CVD_i for county i is defined as the difference between the non-Hispanic (NH) White and Black vaccination rates as below:

$$CVD_i = CVR_{NHWhite,i} - CVR_{Black,i}$$

The Black COVID-19 vaccination rate ($CVR_{Black,i}$) for county i is defined as:

$$CVR_{Black,i} = \frac{NCVacc_{Black,i}}{NRes_{Black,i}}$$

Where: $NCVacc_{Black,i}$ is the number of Black residents in county i receiving at least one COVID-19 vaccine; and $NRes_{Black,i}$ is the number of Black residents in county $i.$

For the non-Hispanic White COVID-19 vaccination rate $CVR_{NHWhite,i}$:

-- for the six states (California, Illinois, New Jersey, Oregon, Texas, and Virginia) that report COVID-19 vaccination data for non-Hispanic (NH) White, $CVR_{NHWhite,i}$ is formally given by:

$$CVR_{NHWhite,i} = \frac{NCVacc_{NHWhite,i}}{NRes_{NHWhite,i}}$$

Where: $NCVacc_{NHWhite,i}$ is the number of non-Hispanic White residents in county i receiving at least one COVID-19 vaccine; and $NRes_{NHWhite,i}$ is the number of non-Hispanic White residents in county $i.$

-- for the ten states that report vaccination data for White and Black people that aggregate ethnicity, only the Vaccination information for total White (instead of non-Hispanic White) is available. We use total White vaccination rate to proxy non-Hispanic White vaccination rate $CVR_{NHWhite,i}$.

Alternative Operationalizations of Disparity. As robustness checks, we also conducted analyses with different operationalizations of disparity, including ratio, log ratio, and scaled absolute disparity. These operationalizations can also be applied to describe the disparities of other variables we will describe below. Their formally definitions are as follows:

- Ratio COVID-19 Vaccination Disparity ($RCVD_i$):

$$RCVD_i = \frac{CVR_{NHWhite,i}}{CVR_{Black,i}}$$

- Logarithm Ratio COVID-19 Vaccination Disparity ($\ln RCVD_i$):

$$\ln RCVD_i = \ln\left(\frac{CVR_{NHWhite,i}}{CVR_{Black,i}}\right)$$

- Scaled Absolute COVID-19 Vaccination Disparity ($SCVD_i$):

$$SCVD_i = \frac{CVR_{NHWhite,i} - CVR_{Black,i}}{Average\ Rate_i},$$

where $AverageRate_i$ is defined as:

$$AverageRate_i = \frac{NCVacc_{Black,i} + NCVacc_{NHWhite,i}}{NRes_{Black,i} + NRes_{NHWhite,i}}$$

Flu Vaccination. We collected county-level data for influenza virus vaccination among Medicare beneficiaries from the CMS Mapping Medical Disparity Tool (22). The latest data reflects behavior from 2019 when flu vaccination was not affected by the COVID-19 pandemic. CMS reports rates of flu vaccination for the non-Hispanic White population and the Black population of any ethnicity.

Flu Vaccination Disparity. Flu vaccination disparity (FVD) was calculated following the same approach as COVID-19 vaccination disparity. Similar to COVID-19, the Black Flu vaccination rate ($FVR_{Black,i}$), and non-Hispanic White Flu vaccination rate ($FVR_{NHWhite,i}$) for county i are calculated as:

$$FVR_{Black,i} = \frac{NFVacc_{Black,i}}{NRes_{Black,i}}$$

Where $NFVacc_{Black,i}$ is the number of Black residents in county i receiving flu vaccine; and $NRes_{Black,i}$ is the number of Black residents in county i.

Similarly,

$$FVR_{NHWhite,i} = \frac{NFVacc_{NHWhite,i}}{NRes_{NHWhite,i}}$$

Where: $NFVacc_{NHWhite,i}$ is the number of non-Hispanic White residents in county i receiving flu vaccine; and $NRes_{NHWhite,i}$ is the number of non-Hispanic White residents in county i.

Flu Vaccination Disparity for county i (FVD_i) is defined as:

$$FVD_i = FVR_{NHWhite,i} - FVR_{Black,i}$$

Different from COVID-19 vaccination data, all 16 states provide vaccination information for non-Hispanic White people.

Predictor Variables

We considered five key dimensions of social determinants of health (23) when determining which measures to include in our model explaining COVID-19 vaccination disparities. For each social determinant of health, we focused on predictors that we expected to be directly related to the impact of COVID-19, factors expected to be associated with vaccination behaviors (general or COVID-specific), and factors that have been associated with racial disparities in health or other life domains. Where the information is available, we model both social determinants for the county overall as well as construct measures of disparity between non-Hispanic Whites and Blacks. Below, we outline the details of how our predictor variables were operationalized.

Economic Stability

Median Income. County-level household median income data were collected from 2019 American Community Survey (ACS) 5-year estimates, which provide information by race (24). Prior research has highlighted the relationship between neighborhood income and a number of health disparities including COVID-19 outcomes (25, 26).

Median Income Disparity. Median income disparity is defined as follows:

$$MedianIncomeDisparity_i = MedianIncome_{NHWhite,i} - MedianIncome_{Black,i}$$

Education Access and Quality

High School Graduation Rate. County-level education attainment data by race were collected from the 5-year ACS estimates (27). We focus on the percentage of population that qualifies as a high school graduate or higher in both races. Education is also considered an important factor in shaping health outcomes and racial disparities in health (23-26).

High School Disparity. Following the same approach of calculating COVID-19 vaccination disparity CVF_i, the Black high school rate ($HighSchoolRate_{Black,i}$), and non-Hispanic White high school rate ($HighSchoolRate_{NHWhite,i}$) for county i are calculated as:

$$HighSchoolRate_{Black,i} = \frac{NHighSchool_{Black,i}}{NRes_{Black,i}}$$

Where $NHighSchool_{Black,i}$ is the number of Black residents in county i achieving high school or higher education attainment; and $NRes_{Black,i}$ is the number of Black residents in county i.

Similarly,

$$HighSchoolRate_{NHWhite,i} = \frac{NHighSchool_{NHWhite,i}}{NRes_{NHWhite,i}}$$

Where $NHighSchool_{NHWhite,i}$ is the number of non-Hispanic White residents in county i achieving high school or higher education attainment; and $NRes_{NHWhite,i}$ is the number of non-Hispanic White residents in county i.

We define High school disparity for county i ($HighSchoolDisparity_i$) as:

$$HighSchoolDisparity_i = HighSchoolRate_{NHWhite,i} - HighSchoolRate_{Black,i}$$

Health Care Access and Quality

COVID-19 Cases per Capita. We collected county level COVID-19 case count data maintained by the Center for Systems Science and Engineering (CSSE) at Johns Hopkins University (28), which is based on information compiled from state and county governments. We focus on cumulative COVID-19 cases in each county and divide this figure by the total population in the county to calculate cases per capita. Greater numbers of COVID-19 cases in a county may be

associated with perceptions of the severity of the disease, which in turn have been shown to predict positive attitudes toward COVID-19 vaccines (29).

Number of Healthcare Facilities per Capita. Given that greater access to vaccines may increase likelihood of vaccination, we collected the number of healthcare facilities in each county that could provide the COVID-19 vaccine from VaxMap 2.0 developed by West Health Policy Center and University of Pittsburgh School of Pharmacy (30). These potential healthcare facilities include: federally qualified healthcare centers, hospital outpatient departments, community pharmacies, and rural health clinics. VaxMap 2.0 gathers information from the National Council for Prescription Drug Programs, the Health Resources and Services Administration, and the Centers for Medicare and Medicaid Services to generate these estimates. We divide the number of healthcare facilities in a county by the total population in that county to calculate number of healthcare facilities per capita.

Neighborhood and Built Environment
Home IT Rate. County-level computer ownership and internet subscription data for each race group were collected from 5-year ACS estimates (31). We model the proportion of the total population that owns any computer (desktop or laptop, smartphone, tablet or other portable wireless computer, or other computer) *and* has broadband internet access. We include this variable because much of the access to COVID-19 vaccines is being driven by online appointments.

Home IT disparity. Similar to calculating COVID-19 vaccination disparity, Black Home IT rate ($HomeITRate_{Black,i}$), and non-Hispanic White Home IT rate ($HomeITRate_{NHWhite,i}$) for county i are as follows:

$$HomeITRate_{Black,i} = \frac{NHomeIT_{Black,i}}{NRes_{Black,i}}$$

Where $NHomeIT_{Black,i}$ is the number of Black residents in county i who own a computer and have broadband internet access; and $NRes_{Black,i}$ is the number of Black residents in county i.

Similarly,

$$HomeITRate_{NHWhite,i} = \frac{NHomeIT_{NHWhite,i}}{NRes_{NHWhite,i}}$$

Where $NHomeIT_{NHWhite,i}$ is the number of non-Hispanic White residents in county i who own a computer and have broadband internet access; and $NRes_{NHWhite,i}$ is the number of non-Hispanic White residents in county i.

We define home IT disparity as:

$$HomeITDisparity_i = HomeITRate_{NHWhite,i} - HomeITRate_{Black,i}$$

Urban. We included a dummy-coded variable representing whether a county is considered urban or rural according to data collected from the CMS Mapping Medicare Disparities Tool (22). This

classification designates a county as urban if the area is metropolitan (a core urban area of 50,000 or more population) or micropolitan (an urban core of at least 10,000 but less than 50,000 population). All other counties are considered rural.

Rate of Vehicle Ownership. We collected county-level household vehicle ownership data from the CDC Social Vulnerability Index from year 2018 (32, 33). We take the inverse of the original data, which was coded as the proportion of *no* vehicle ownership. These data do not include rate of vehicle ownership by race, therefore only the overall rate is included in our analysis. Vehicle ownership is included because it may be important for transportation to vaccination sites.

Social and Community Context

Political Ideology. To model political ideology, we used the share of votes cast for the Republican candidate (President Donald J. Trump) in the 2020 election collected from USA Today (34). We use Republican vote share because survey evidence suggests that political affiliation is an important predictor of attitudes toward COVID-19 and the COVID-19 vaccine in particular (35). This approach is also consistent with prior research that showed Democratic counties were more likely to reduce mobility in response to stay-at-home-orders for COVID-19 prevention, which used the vote margin in favor of President Trump as a predictor (36).

Segregation Index. We collected Black-White segregation index measures from the 2021 County Health Rankings (37). Based on data from ACS 2015 to 2019, the residential segregation index ranges from 0 (complete integration) to 100 (complete segregation). These values represent the percentage of either Black or White residents who would have to move to yield a distribution that is similar to that of the larger area. Segregation in communities has been associated with a number of important racial disparities in health (38).

Racial Bias. We modeled implicit racial bias using estimates published in prior research (39), which are based on responses collected from Project Implicit, a website where individuals voluntarily complete psychological attitude measures (40). We used the weighted measure of implicit bias, which accounts for the number of observations available for each county. Measures of implicit bias assess the automatic association between race (White and Black) and attitudes (good or bad), with larger values indicating greater bias against Blacks. Such measures of bias have previously been linked to racial disparities in school disciplinary actions (39).

<u>Covariates</u>

Vaccine Hesitancy. We collected county level COVID-19 vaccine hesitancy data from the Department of Health and Human Services, Office of the Assistant Secretary for Planning and Evaluation (41). These data include responses to Phase 3 of the Household Pulse Survey (March 17-March 29, 2021) question "*Once a vaccine to prevent COVID-19 is available to you, would you get a vaccine*?". Participants could respond with the following options: 1) "definitely get a vaccine", 2) "probably get a vaccine", 3) "probably not get a vaccine", and 4) "definitely not get a vaccine". Those who respond that they will "probably not" or "definitely not" get a vaccine are considered vaccine hesitant. Anyone who reported already receiving a COVID-19 vaccine in a prior question or responded that they would "probably" or "definitely" get a vaccine are considered *not* vaccine hesitant. The data also reflect weighting according to ACS demographic estimates. These data were not available by race, therefore we model only overall vaccine hesitancy.

Proportion of Black Residents. The proportion of Black residents was extracted from demographic data published by the Census Bureau (21).

Proportion of Population Age 75 and Above and Disparity in Population Age 75 and Above. We collected the proportion of the population aged 75 or older from the same data source (21). We then calculated the difference in the proportion of adults aged 75 or older by race. Similar to calculating COVID-19 vaccination disparity, the Black age 75 and above rate ($Above75Rate_{Black,i}$), and non-Hispanic White age 75 and above rate ($Above75Rate_{NHWhite,i}$) for county i are as follows:

$$Above75Rate_{Black,i} = \frac{NAbove75_{Black,i}}{NRes_{Black,i}}$$

Where $NAbove75_{Black,i}$ is the number of Black residents in county i of ages 75 or older; and $NRes_{Black,i}$ is the number of Black residents in county $i.$

Similarly,

$$Above75Rate_{NHWhite,i} = \frac{NAbove75_{NHWhite,i}}{NRes_{NHWhite,i}}$$

Where $NAbove75_{NHWhite,i}$ is the number of non-Hispanic White residents in county i of ages 75 or older; and $NRes_{NHWhite,i}$ is the number of non-Hispanic White residents in county $i.$

We define the age 75 and above disparity ($Above75Disparity_i$) as:

$$Above75Disparity_i = Above75Rate_{NHWhite,i} - Above75Rate_{Black,i}$$

Proportion of Remaining Eligible Population. The demographic data available only provides population estimates only for 5-year age bands (21), thus we could not collect exact data for the 16+ population reflecting the cut-off for the Pfizer vaccine nor the 18+ population reflecting the cut-off for Moderna and the Johnson and Johnson vaccines. Leveraging the information available, we use the proportion of the population aged 15-74 to account for the number of individuals eligible for the vaccine (complementing estimates of the population 75+). We then calculated the difference in the proportion of populated 15-74 years of age by race. Similar to calculating COVID-19 vaccination disparity, Black age 15 to 74 rate ($Age15To74Rate_{Black,i}$), and non-Hispanic White age 15 to 74 rate ($Age15To74Rate_{NHWhite,i}$) for county i are given as follows:

$$Age15To74Rate_{Black,i} = \frac{NAge15To74_{Black,i}}{NRes_{Black,i}}$$

Where $NAge15To74_{Black,i}$ is the number of Black residents in county i of ages 15 to 74 (inclusive); and $NRes_{Black,i}$ is the number of Black residents in county $i.$

Similarly,

$$Age15To74Rate_{NHWhite,i} = \frac{NAge15To74_{NHWhite,i}}{NRes_{NHWhite,i}}$$

Where $NAge15To74Rate_{NHWhite,i}$ is the number of non-Hispanic White residents in county i of ages 15 to 74 (inclusive); and $NRes_{NHWhite,i}$ is the number of non-Hispanic White residents in county i.

We define the age 15-to-74 disparity as:

$$Age15To74Disparity_i = Age15To74Rate_{NHWhite,i} - Age15To74Rate_{Black,i}$$

Recent Positivity Rate. In addition to cumulative COVID-19 case count, recent positivity rates may influence perceived threat of COVID-19 and, in turn, vaccination behavior. We collected average daily positivity rate for Nucleic Acid Amplification Tests (NAAT) administered from April 12, 2021-April 19, 2021 from the CDC (42).

Analytic Approach

Data Preprocessing. Our initial dataset consisted of 1293 counties from 16 US states. However, some counties within those 16 states only report vaccination statistics for one race, so we were unable to measure vaccination disparity in those counties. We excluded those counties, resulting in a sample of 1203 counties. We also excluded counties whose COVID-19 vaccination rates for Black and/or White residents exceeded 100%, yielding 1186 counties. We then excluded observations with missing values for any dependent or predictor variables, which led to 776 counties. In some counties, there are very few Black residents; for example, according to the 2019 ACS 5-year estimate, there are 9 Black people in Kent, Texas while the population of White people is relatively large. To increase the robustness of the measurement of vaccine disparity, we excluded counties where the proportion of Black residents is very small. The cutoff for exclusion was set at 273 Black residents, which is the first quartile of the Black population across all counties in the initial 1293 counties sample. Following this exclusion, our final sample for regression analysis consists of 756 counties.

Methods for Regression analysis.[1] The main analyses in the context are performed using OLS regressions: one with COVID-19 disparity (CVD) and a second with flu vaccination disparity (FVD) among Blacks and Whites as the dependent variable. In the regression models, in addition to the main explanatory variables, we include state dummy variables to account for state heterogeneity in vaccination policies, since states have slightly different COVID-19 vaccination eligibility in terms of age and occupations (44). Before conducting the regression analysis, all variables except the dependent variables and dummy variables were standardized to yield mean = 0 and standard deviation = 1. Furthermore, to alleviate the influence of outliers, we winsorized all variables at 5th and 95th percentiles. Finally, the regression models were weighted using total county population, with robust standard errors clustered at the state level.

[1] https://github.com/CHIDS-UMD/Covid19-Vaccination-Race-Disparity-Publish#main-reg

$$
\begin{aligned}
\{CVD_i, FVD_i\} = \; & \beta_0 + \beta_1 MedianIncome_i + \beta_2 MedianIncomeDisparity_i \\
& + \beta_3 HighSchoolGraduationRate_i + \beta_4 HighSchoolDisparity_i \\
& + \beta_5 HealthFacilitiesPerCapita_i + \beta_6 COVIDCasesPerCapita_i + \beta_7 HomeITRate_i \\
& + \beta_8 HomeITDisparity_i + \beta_9 Urban_i + \beta_{10} RateOfVehicleOwnership_i \\
& + \beta_{11} PoliticalIdeology_i + \beta_{12} SegregationIndex_i + \beta_{13} RacialBias_i \\
& + \beta_{14} VaccineHesitancy_i + \beta_{15} PropofBlackPopulation_i + \sum_{j=1}^{15} StateDummy_{ij} + \varepsilon_i
\end{aligned}
$$

Extended Methods for Robustness Checks

Below are the details of the robustness checks reflected in the main context. The code and data are accessible via the GitHub URL in each section's footnote.

Earlier Time Periods.[2] We developed codes to conduct robustness checks using COVID-19 vaccination disparity data collected at two earlier time periods, March 27 and April 7.

Controlling for Flu Vaccination Rates.[3] As a further robustness check, we controlled for flu vaccination rates and disparity in flu vaccination rates. While we already control for overall vaccine hesitancy in our main analysis, the addition of flu vaccination rates allows us to capture non-COVID-19 specific vaccination behavior and observed disparities by race. Of note, the flu vaccination data from 2019 comes from a Medicare population.

Controlling for Older Adult Population.[4] While continuing to include flu vaccination measures, we then controlled for the proportion of adults aged 75 or older and racial differences in the rate of older adults. This is because in the early stage of vaccination, older adults were given higher priority for vaccination, which might affect racial disparity due to the longer life expectancy of Whites (45, 46).

Controlling for Vaccine Eligible Population.[5] In addition to differences in the priority populations of adults above age 75, we also added a variable to control for proportion of the remaining population eligible for the vaccines. We approximate the eligible population by controlling for a county's population aged 15-74 (and related disparity) in one set of analyses and the population aged 20-74 (and related disparity) in a second.

Defining Rates by Eligible Population.[6] Complementing the approach of adding additional age controls, we also performed robustness checks that defined vaccination rates relative to the

---

[2] https://github.com/CHIDS-UMD/Covid19-Vaccination-Race-Disparity-Publish#different-dates-and-full-vaccination-rate-types

[3] https://github.com/CHIDS-UMD/Covid19-Vaccination-Race-Disparity-Publish#different-age-group-controls

[4] https://github.com/CHIDS-UMD/Covid19-Vaccination-Race-Disparity-Publish#different-age-group-controls

[5] https://github.com/CHIDS-UMD/Covid19-Vaccination-Race-Disparity-Publish#different-age-group-controls

[6] https://github.com/CHIDS-UMD/Covid19-Vaccination-Race-Disparity-Publish#different-age-group-controls

population above age 15 in a county and the population above age 20. This is in contrast to the approach of defining vaccination rates relative to the population of the entire county.

Alternative Operationalizations of Disparity.[7] To assess the robustness of our findings when operationalizing disparity in different ways, we calculated vaccination disparity as a ratio of the White vaccination rate compared to the Black vaccination rate (White Vaccination Rate/Black Vaccination Rate). In addition, we treated predictor variables modeling disparity (e.g., high school graduation rate disparity, median income disparity, etc.) following the same ratio method.

In addition to defining disparity as a ratio, we performed additional analyses defining disparity scaled relative to the overall vaccination rate in a county (i.e., $SCVD_i$). In this model, we adopt a similar scaled approach to define disparities in our predictor variables where applicable.

Residential Mobility.[8] Some areas saw high levels of mobility during the pandemic, which may bias estimates of vaccination rates. To address this concern, we identified a source that provided information about the areas that saw the greatest movement during the pandemic based on data from 75,000 moves (47). The list includes 10 cities with the greatest net increase in movement and the 10 cities with the greatest net decrease in movement, some of which are not represented in the counties included in our analysis. We exclude the 12 relevant counties represented in our data for this additional robustness checks.

At Least One and Full Vaccination Results on May 20, 2021.[9] We also collected recent full vaccination rate data from May 20, 2021 to explore whether our findings hold when including second dose data. Using these data, we replicate our main analysis approach and robustness checks. We note that fewer states report full vaccination rates by race, yielding a final sample of 534 counties for the full vaccination rate analyses.

Addressing Multicollinearity.[10] To address multicollinearity of variables, we examined the variance inflation factors (VIF) for the variables in our main models. We found that the only variables that exceeded the conventional cut-off for VIFs of 10 were several state dummy variables and vaccine hesitancy. To investigate the impact of this multicollinearity, we ran an additional robustness check excluding vaccine hesitancy from the model.

Treatment of Ethnicity.[11] Given the different treatments of ethnicity in the data made available, we conducted further robustness checks by splitting our sample into two subgroups, those that provide estimates for non-Hispanic White vaccination rates specifically (six states) and those that do not (ten states).

Consistency with our Original Submission.[12] To further validate the robustness of our finding, we also use the approach in our original submission to calculate COVID-19 vaccination

[7] https://github.com/CHIDS-UMD/Covid19-Vaccination-Race-Disparity-Publish#different-disparity-operationalizations

[8] https://github.com/CHIDS-UMD/Covid19-Vaccination-Race-Disparity-Publish#residential-mobility

[9] https://github.com/CHIDS-UMD/Covid19-Vaccination-Race-Disparity-Publish#different-dates-and-full-vaccination-rate-types

[10] https://github.com/CHIDS-UMD/Covid19-Vaccination-Race-Disparity-Publish#avoid-collinearity-by-droping-hesitancy-variable

[11] https://github.com/CHIDS-UMD/Covid19-Vaccination-Race-Disparity-Publish#subsample-analysis-6-states-and-10-states

[12] https://github.com/CHIDS-UMD/Covid19-Vaccination-Race-Disparity-Publish#vaccination-rate-on-whole-white-population

disparities, where the White vaccination rate is based on total White population rather than Non-Hispanic White population for all states in our sample.

Reproducibility

All data and code are available in a publicly accessible GitHub repository available here: https://github.com/CHIDS-UMD/Covid19-Vaccination-Race-Disparity-Publish (43).

References

1. State of California, Vaccination progress data. https://covid19.ca.gov/vaccination-progress-data/. Accessed 19 April 2021.
2. Illinois Department of Public Health, COVID-19 Vaccine Administration Data. https://www.dph.illinois.gov/covid19/vaccinedata?county=Illinois. Accessed 19 April 2021.
3. Indiana State Department of Health, Novel Coronavirus: Vaccine Dashboard. https://www.coronavirus.in.gov/vaccine/2680.htm. Accessed 19 April 2021.
4. State of Maine, State of Maine COVID-19 Vaccination Dashboard. http://www.maine.gov/covid19/vaccines/dashboard. Accessed 19 April 2021.
5. NJ Health, New Jersey COVID-19 Dashboard. https://www.nj.gov/health/cd/topics/covid2019_dashboard.shtml. Accessed 19 April 2021.
6. New York State, COVID-19 Vaccine Tracker. https://covid19vaccine.health.ny.gov/covid-19-vaccine-tracker. Accessed 19 April 2021.
7. North Carolina Department of Health and Human Services, Data Behind the Dashboards. https://covid19.ncdhhs.gov/dashboard/data-behind-dashboards. Accessed 19 April 2021.
8. Ohio Department of Health, COVID-19 Vaccination Dashboard. https://coronavirus.ohio.gov/wps/portal/gov/covid-19/dashboards/covid-19-vaccine/covid-19-vaccination-dashboard. Accessed 19 April 2021.
9. Oregon Health Authority, Oregon COVID-19 Vaccination Trends. https://public.tableau.com/profile/oregon.health.authority.covid.19#!/vizhome/OregonCOVID-19VaccinationTrends/OregonCountyVaccinationTrends. Accessed 19 April 2021.
10. Pennsylvania Department of Health, COVID Vaccines. https://www.health.pa.gov/topics/disease/coronavirus/Vaccine/Pages/Vaccine.aspx. Accessed 19 April 2021.
11. South Carolina Department of Health and Environmental Control, COVID-19 Vaccination Dashboard. https://scdhec.gov/covid19/covid-19-vaccination-dashboard. Accessed 19 April 2021.
12. Tennessee Department of Health, Downloadable Datasets. https://www.tn.gov/health/cedep/ncov/data/downloadable-datasets.html. Accessed 19 April 2021.
13. Texas Health and Human Services, COVID-19 Vaccine in Texas. https://tabexternal.dshs.texas.gov/t/THD/views/COVID-19VaccineinTexasDashboard/Summary?%3Aorigin=card_share_link&%3Aembed=y&%3AisGuestRedirectFromVizportal=y. Accessed 19 April 2021.

14. Virginia Department of Health, COVID-19 Vaccine Demographics. https://www.vdh.virginia.gov/coronavirus/covid-19-vaccine-demographics/. Accessed 19 April 2021.
15. West Virginia Department of Health & Human Resources, Coronavirus Disease 2019. https://dhhr.wv.gov/COVID-19/Pages/default.aspx. Accessed 19 April 2021.
16. Wisconsin Department of Health Services, COVID-19: Vaccine Data. https://www.dhs.wisconsin.gov/covid-19/vaccine-data.htm#day. Accessed 19 April 2021.
17. H. B. Demeke, A. S. Johnson, B. Wu, R. Moonesinghe, H. D. Dean, Unequal declines in absolute and relative disparities in HIV diagnoses among Black women, United States, 2008 to 2016. *Am. J. Public Health* **108**, S299–S303 (2018).
18. S. McMorrow, S. K. Long, G. M. Kenney, N. Anderson, Uninsurance disparities have narrowed for Black and Hispanic adults under the Affordable Care Act. *Health Aff.* **34**, 1774–1778 (2015).
19. A. McBride, *et al.*, National survey on the effect of oncology drug shortages on cancer care. *Am. J. Health. Syst. Pharm.* **70**, 609–617 (2013).
20. E. C. Schneider, P. D. Cleary, A. M. Zaslavsky, A. M. Epstein, Racial disparity in influenza vaccination: Does managed care narrow the gap between African Americans and Whites? *JAMA* **286**, 1455–1460 (2001).
21. The United States Census Bureau, County Population by Characteristics: 2010-2019. https://www.census.gov/data/tables/time-series/demo/popest/2010s-counties-detail.html. Accessed 25 March 2021.
22. Centers for Medicare and Medicaid Services, Mapping Medicare disparities tool. https://data.cms.gov/mapping-medicare-disparities. Accessed 25 March 2021.
23. Health and Human Services Office of Disease Prevention and Health Promotion, Social Determinants of Health - Healthy People 2030. https://health.gov/healthypeople/objectives-and-data/social-determinants-health. Accessed 1 June 2021.
24. The United States Census Bureau, Income in the Past 12 Months (in 2019 inflation-adjusted dollars). https://data.census.gov/cedsci/table?q=income%20county&g=0100000US.050000&tid=ACSST5Y2019.S1901&hidePreview=true. Accessed 25 March 2021.
25. J. F. Figueroa, R. K. Wadhera, D. Lee, R. W. Yeh, B. D. Sommers, Community-Level Factors associated with racial and ethnic disparities in COVID-19 rates in Massachusetts. *Health Affairs* **39**, 1984–1992 (2020).
26. C. J. L. Murray, *et al.*, Eight Americas: Investigating Mortality Disparities across Races, Counties, and Race-Counties in the United States. *PLOS Medicine* **3**, e260 (2006).
27. The United States Census Bureau, Educational attainment. https://data.census.gov/cedsci/table?q=Education%20Attainment&tid=ACSST5Y2019.S1501&hidePreview=false. Accessed 25 March 2021.
28. Johns Hopkins University Center for Systems Science and Engineering, COVID-19 data repository. https://github.com/CSSEGISandData/COVID-19. Accessed 25 March 2021.
29. P. L. Reiter, M. L. Pennell, M. L. Katz, Acceptability of a COVID-19 vaccine among adults in the United States: How many people would get vaccinated? *Vaccine* **38**, 6500–6507 (2020).

30. L. A. Berenbrok, *et al.*, Access to Potential COVID-19 Vaccine Administration Facilities: A Geographic Information Systems Analysis. https://www.westhealth.org/resource/vaxmap-potential-covid-19-vaccine-locations/. Accessed 25 March 2021.
31. The United States Census Bureau, Types of computer and internet subscription. https://data.census.gov/cedsci/table?q=ACSST5Y2019.S2802_2021-03-20T111825&tid=ACSST1Y2019.S2802. Accessed 25 March 2021.
32. B. E. Flanagan, E. W. Gregory, E. J. Hallisey, J. L. Heitgerd, B. Lewis, A Social Vulnerability Index for Disaster Management. *J. Homel. Secur. Emerg. Manag.* **8** (2011).
33. Center for Disease Control and Prevention, Social Vulnerability Index (SVI). https://www.atsdr.cdc.gov/placeandhealth/svi/index.html. Accessed 25 March 2021.
34. M. Thorson, J. Haseman, C. Procell, Four maps that show how America voted in the 2020 election with results by county, number of voters, 29 December 2020. https://www.usatoday.com/in-depth/graphics/2020/11/10/election-maps-2020-america-county-results-more-voters/6226197002/. Accessed 25 March 2021.
35. C. Funk, A. Tyson, Growing share of Americans say they plan to get a COVID-19 vaccine – or already have. Pew Research Center, 5 March 2021. Accessed 22 April 2021.
36. G. Grossman, S. Kim, J. M. Rexer, H. Thirumurthy, Political partisanship influences behavioral responses to governors' recommendations for COVID-19 prevention in the United States. *Proc Natl Acad Sci USA* **117**, 24144–24153 (2020).
37. County Health Rankings, Residential segregation - Black/White. https://www.countyhealthrankings.org/explore-health-rankings/measures-data-sources/county-health-rankings-model/health-factors/social-and-economic-factors/family-social-support/residential-segregation-blackwhite. Accessed 25 March 2021.
38. M. R. Kramer, C. R. Hogue, Is segregation bad for your health? *Epidemiologic Reviews* **31**, 178–194 (2009).
39. T. Riddle, S. Sinclair, Racial disparities in school-based disciplinary actions are associated with county-level rates of racial bias. *Proc. Natl. Acad. Sci.* **116**, 8255–8260 (2019).
40. K. Xu, B. Nosek, A. Greenwald, Psychology data from the race Implicit Association Test on the Project Implicit demo website. *J. Open Psychol. Data* **2**, e3 (2014).
41. Health and Human Services Office of the Assistant Secretary for Planning and Evaluation. Vaccine hesitancy for COVID-19: State, county, and local estimates. https://aspe.hhs.gov/pdf-report/vaccine-hesitancy. Accessed 16 April 2021.
42. CDC, COVID Data Tracker. Integrated County View. Centers for Disease Control and Prevention. Accessed 1 June 2021.
43. R. Agarwal, *et al.*, Socioeconomic privilege and political ideology are associated with racial disparity in COVID-19 vaccination methods and materials. https://github.com/CHIDS-UMD/Covid19-Vaccination-Race-Disparity-Publish. Deposited 1 July 2021.
44. The New York Times, See How the Vaccine Rollout Is Going in Your County and State. https://www.nytimes.com/interactive/2020/us/covid-19-vaccine-doses.html. Accessed 22 April 2021.
45. E. Arias, B. Tejada-Vera, F. Ahmad, Provisional Life Expectancy Estimates for January through June, 2020. *Vital Statistics Rapid Release*, **10**. Hyattsville, MD: National Center for Health Statistics. February 2021.

46. E. Wrigley-Field, US racial inequality may be as deadly as COVID-19. *Proc. Natl. Acad. Sci.* **117**, 21854–21856 (2020).
47. HireAHelper. Where are people moving to during COVID-19, in 2020 and beyond? Accessed 1 June 2021.
